# Assessment of nanomaterial-induced hepatotoxicity using a 3D human primary multi-cellular microtissue exposed repeatedly over 21 days - the suitability of the in vitro system as an in vivo surrogate

**DOI:** 10.1186/s12989-019-0326-0

**Published:** 2019-11-19

**Authors:** Ali Kermanizadeh, Trine Berthing, Ewa Guzniczak, Melanie Wheeldon, Graeme Whyte, Ulla Vogel, Wolfgang Moritz, Vicki Stone

**Affiliations:** 1 Heriot Watt University, School of Engineering and Physical Sciences, Edinburgh, UK; 2 National Research Centre for the Working Environment, Copenhagen, Denmark; 3 InSphero AG, Wagistrasse 27, Schlieren, Switzerland

**Keywords:** 3D primary human multi-cellular liver microtissue, Kupffer cells, In vitro hepatotoxicology, In vitro vs. in vivo comparisons

## Abstract

**Background:**

With ever-increasing exposure to engineered nanomaterials (NMs), there is an urgent need to evaluate the probability of consequential adverse effects. The potential for NM translocation to distal organs is a realistic prospect, with the liver being one of the most important target organs. Traditional in vitro or ex vivo hepatic toxicology models are often limiting (i.e. short life-span, reduced metabolic activity, lacking important cell populations, etc.). In this study, we scrutinize a 3D human liver microtissue (MT) model (composed of primary hepatocytes and non-parenchymal cells). This unique experiment benefits from long-term (3 weeks) repeated very low exposure concentrations, as well as incorporation of recovery periods (up to 2 weeks), in an attempt to account for the liver’s recovery capacity in vivo. As a means of assessing the toxicological potential of NMs, cell cytotoxicity (cell membrane integrity and aspartate aminotransferase (AST) activity), pro/anti-inflammatory response and hepatic function were investigated.

**Results:**

The data showed that 2 weeks of cell culture might be close to limits before subtle ageing effects start to overshadow low sub-lethal NM-induced cellular responses in this test system (adenylate kinase (AK) cytotoxicity assay). We showed that in vitro AST measurement are not suitable in a nanotoxicological context. Moreover, the cytokine analysis (IL6, IL8, IL10 and TNF-α) proved useful in highlighting recovery periods as being sufficient for allowing a reduction in the pro-inflammatory response. Next, low soluble NM-treated MT showed a concentration-dependent penetration of materials deep into the tissue.

**Conclusion:**

In this study the advantages and pitfalls of the multi-cellular primary liver MT are discussed. Furthermore, we explore a number of important considerations for allowing more meaningful in vitro vs. in vivo comparisons in the field of hepatic nanotoxicology.

## Background

The rapid expansion in the commercial use of engineered nanomaterials (NMs) in various industries has led to increasing interest in the fields of nanotechnology and nanomedicine [1, 2]. The ever-increasing manufacture and utilisation of NMs enhances the need to assess the potential hazard and safety concerns to those encountering these materials [3–5].

The liver has been identified as an extremely important organ for the accumulation and potentially the clearance of NMs from the bloodstream [6–10]. In addition to occupational and consumer exposure via inhalation and ingestion, recent advances in the field of nanomedicine mean that direct entry of NMs into the bloodstream is a very real prospect [1–6].

The liver is the bodies’ principal detoxification centre, removing foreign substances and waste products i.e. bilirubin [11]. The organ is characterised by its distinct populations of cells, amongst which the hepatocytes (parenchymal cells), the resident macrophage population (Kupffer cells - KCs) and sinusoidal endothelial cells are amongst some of the most important [12–14]. In particular and of great importance in particle hepatotoxicity is the fact that the KCs line the liver sinusoids. This locality means that these cells have constant contact with any materials or organisms, which reach the organ via the bloodstream (including from the gastrointestinal tract). Additionally, once activated KCs are the most important cell populations in the modulation and governance of overall hepatic immune response in the non-diseased organ [14, 15]. However, the KCs are not likely to be 100% effective at prevention of the hepatocytes encountering materials as very small NMs could access the parenchymal cells via the open fenestrations in the liver sinusoids. In a recent study in which mice were dosed by intravenous injection or intratracheal injection, both CeO_2_ and TiO_2_ NM aggregates were identified in the sinusoids and often in the KCs up to 180 days post exposure [10].

Despite the significant and notable improvements to hepatic in vitro model systems over the last 5 years, the majority of the models still have some major limitations, which need to be fully considered. It is generally accepted that it is often difficult to make direct or meaningful comparisons between in vitro and in vivo hepatic responses. One of the key reasons for the lack of comparability between biological responses is that they are rarely like for like. For example, mono-cultures lack cross-talk between different cell types and between different organs. It is also difficult to identify in vivo endpoints that are measurable in vitro. For example, measuring cytotoxicity in vitro will rarely be sufficient to equate or compare to an in vivo toxicity response (i.e. difficulties in identifying key in vivo biomarkers that can be measured in vitro). Of particular importance and unique for the liver, is the inability of in vitro models to emulate the liver’s incredible regeneration capability. The livers ability to regenerate is critical in disease recovery, and so is important in distinguishing between the ability of different NMs to induce acute versus longer-term harm to the human liver. This consideration of liver recovery is therefore imperative for in vitro NM hazard assessment.

In an attempt to address the above mentioned issues, this study was designed to scrutinize a scaffold free, 3D liver microtissue (MT) spheroid model, composed of primary human hepatocytes and primary human liver-derived non-parenchymal cells (NPC) (KCs and sinusoidal endothelial cells). In these experiments, the MT were exposed to three different NMs with different physicochemical properties - namely: zinc oxide (ZnO), titanium dioxide (TiO_2_), and cerium dioxide (CeO_2_) and the positive control quartz containing ~ 87% crystalline silica (DQ12) (selected from panel of materials utilised in H2020 funded PATROLS project [16]). These materials are representative of NMs currently incorporated in consumer products including sunscreens, cosmetics, clothing and sporting goods [2]. In addition, to the utilisation of a physiologically relevant multi-cellular human primary cell hepatic test system, this unique experiment benefits from long-term (3 weeks) very low dosing, as well as incorporation of recovery periods (up to 2 weeks), in an attempt to account for the organs recovery capacity in vivo. Moreover, in previous in vivo examinations, in which an NM intratracheal instillation dose of 162 μg/mouse (corresponding to pulmonary occupational exposure of 14 working days) showed the translocated doses of 1–2 μg/g liver after 180 days [17]. This 1 μg/g in vivo NM dose corresponds to a dose of 1 μg/ml in this in vitro experiment highlighting the biological relevance of the low concentrations utilised in this study. The dosing considerations are based on previous in vivo data demonstrating that 1–2% of total administered NP dose following inhalation translocate into the bloodstream [4]. This was followed by the assumption that 100% NPs in bloodstream reach the liver. As a means of assessing the toxicological potential of NMs, cell cytotoxicity (cell membrane integrity and aspartate aminotransferase (AST) activity), pro/anti-inflammatory response (cytokine secretion) and hepatic function (albumin production) were investigated. Moreover, the cell-NM interaction on the surface of the spheroids as well as internalisation and penetration of materials into the core of the MT was scrutinised.

## Methods

### Liver MT maintenance

The study utilized 3D InSight™ human liver MT, composed of multi-donor primary hepatocytes in co-culture with NPC containing primary KCs and primary liver endothelial cells (hepatocyte lot IPHH_17, NPC lot IPHN_08) (InSphero AG, Switzerland). A total of eleven MT plates (96 wells/plate) were utilized. The human liver MT was maintained in complete medium (3D InSight™ human liver maintenance medium - AF (InSphero AG, Switzerland)) at 37 °C, 5% CO_2_, 95% humidity with the medium exchanged (70 μl per well) on the day of arrival and every 2 days thereafter (50 μl per well).

### Nanomaterials

The NMs were sourced as follows: TiO_2_ (JRCNM01005a - NM 105, JRC Nanomaterials Repository - Italy), ZnO (JRCNM01101a - NM 111, JRC Nanomaterials Repository - Italy), CeO_2_ (NM 212, JRC Nanomaterials Repository - Italy) and crystalline silica (DQ12 from Institute of Occupational Medicine - UK). The NMs were sub-sampled under Good Laboratory Practice conditions and preserved under argon in the dark until use (with the exception of the DQ12).

### Characterisation of the panel of NMs

A summarised list of the measured physical and chemical properties of the selected NMs has been re-produced from previous work [18–20]. Furthermore, the hydrodynamic size distributions of the NMs dispersed in complete cell culture medium were determined at a concentration of 10 μg/ml by Dynamic Light Scattering (DLS) using a Zetasizer Nano-ZS (Malvern, USA) Additional file 1. Finally, a Pierce LAL Chromogenic Endotoxin Quantitation Kit (Thermo Scientific, UK) was utilized to test for possible endotoxin contaminations of the tested NMs. The kit was used according to the manufacturer’s guidelines.

### NM dispersion and treatment

NMs were prepared following the NANOGENOTOX dispersion protocol [21]. Briefly, following the sonication step, the stock solution of the materials were immediately transferred to ice before being diluted in complete medium just prior to the experiments. In this experiment, five NM concentrations were used: 0.62, 1.25, 2.5, 5 and 10 μg/ml (total volume of 50 μl added to each well) as well as negative (cell culture medium) and positive (1% Triton-X) (Sigma, UK) controls. For each treatment, five wells were used and experiments were repeated on three separate occasions. The material treatment regimes in the three-week exposure experiments (including recovery periods) are summarised in Table 1. To avoid potential complications with ageing of tissue between different plates, the three NM treatment repetitions were carried out on the same day (morning, lunchtime and late afternoon - with a fresh batch of NMs prepared prior for each exposure). Finally, all concentrations are expressed as μg/ml. This decision is principled on the fact the liver cells are clustered in spheroids and expressing the treatments as μg/cm^2^ would not be appropriate. In addition, the wells are v-shaped so the settling dynamics are likely to be different to a conventional well format.

**Table 1:** NM treatment and toxicological end-points measured in the repeated exposure experiments over a period of 3 weeks

Experiment A	End-points investigated
Material dosing **1** at day 0	–
Medium replacement at **day 1** 24 h recovery period	**AK assay, AST**
Material dosing **2** at day 2	–
Medium replacement at **day 3** 24 h recovery period	**Cytokine secretion, albumin**
Material dosing **3** at day 4	–
Medium replacement at **day 5** 24 h recovery period	**AK assay**
Material dosing **4** at day 6	
Medium replacement at **day 7**	**Cytokine secretion, albumin**
2 weeks recovery (medium change every other day) - collect supernatant **day 21**	**AK assay, cytokine secretion**

Experiment B	End-points investigated
Material dosing **1** at day 0	–
Medium replacement at **day 1** 24 h recovery period	–
Material dosing **2** at day 2	–
Medium replacement at **day 3** 24 h recovery period	–
Material dosing **3** at day 4	–
Medium replacement at **day 5** 24 h recovery	–
Material dosing **4** at day 6	–
Medium replacement at **day 7** 24 h recovery	
Material dosing **5** at day 8	–
Medium replacement at **day 9** 24 h recovery	**Albumin, AST**
Material dosing **6** at day 10	
Medium replacement at **day 11** 24 h recovery	**AK assay**
Material dosing **7** at day 12	Treatment missed
Medium replacement at **day 13** 24 h recovery	
Material dosing **8** at day 14	
Medium replacement at **day 15**	**Cytokine secretion, albumin**
1 week recovery (medium change every other day) - collect supernatant **day 21**	**AK assay, cytokine secretion**

Experiment C	End-points investigated
Material dosing **1** at day 0	–
Medium replacement at **day 1** 24 h recovery period	–
Material dosing **2** at day 2	–
Medium replacement at **day 3** 24 h recovery period	–
Material dosing **3** at day 4	–
Medium replacement at **day 5** 24 h recovery	–
Material dosing **4** at day 6	–
Medium replacement at **day 7** 24 h recovery	–
Material dosing **5** at day 8	–
Medium replacement at **day 9** 24 h recovery	–
Material dosing **6** at day 10	–
Medium replacement at **day 11** 24 h recovery	–
Material dosing **7** at day 12	Treatment missed
Medium replacement at **day 13** 24 h recovery	
Material dosing **8** at day 14	–
Medium replacement at **day 15** 24 h recovery	
Material dosing **9** at day 16	
Medium replacement at **day 17** 24 h recovery	**AST**
Material dosing **10** at day 18	
Medium replacement at **day 19** 24 h recovery	**AK assay, albumin**
Material dosing **11** at day 20	
Collect supernatant **day 21**	**AK assay, cytokine secretion, AST**

### Adenylate kinase (AK) assay

The loss of cell membrane integrity was evaluated utilising a ToxiLight™ bioassay kit (Lonza, USA). Briefly, 20 μl of cell supernatant was transferred to a luminescence compatible plate before the addition of 80 μl of AK detection buffer. The plates were incubated for 5 min at room temperature and luminescence quantified.

### AST quantification

Due to the importance of AST measurements both in the diagnosis of liver disease clinically and as an in vivo biomarker of liver damage in experimental settings, two different methodologies were utilized in an attempt to quantify the enzyme activity in the supernatant of MT over time (as a means of assessing the suitability of this specific end-point in in vitro nanotoxicological experiments). For both methodologies, the supernatant from five MT was pooled and stored at − 80 °C until required.

In the first method, an AST reagent was used in conjunction with UniCel® DxC 800 system and SYNCHRON® Systems Enzyme Validator Set for the determination of AST enzyme activity in the supernatant of the MT. In the assay reaction, the AST catalyses the reversible transamination of L-aspartate and α-ketoglutarate to oxaloacetate and L-glutamine. The oxaloacetate is then reduced to malate in the presence of malate dehydrogenase with the concurrent oxidation of β-Nicotinamide Adenine Dinucleotide.

In the second method, AST enzyme concentrations were determined using an AST ELISA Kit (Novus Biologicals, USA) according to the manufacturer’s instructions.

### Cytokine secretion

The levels of human interleukin (IL)6, IL8, IL10 and tumour necrosis factor-α (TNF-α) secreted from the MT was determined in the cell supernatant using R&D Systems magnetic Luminex® Performance Assay multiplex kits (bead based immunoassay; Bio-techne, USA) according to the manufacturer’s instructions. The protein concentrations were evaluated via a Bio-Rad® Bio-Plex® MAGPIX multiplex reader. The technology is based on the use of analyte-specific antibodies pre-coated onto magnetic microplates embedded with fluorophores at set ratios for each unique microparticle region.

### Albumin production

After exposure, the supernatants (from both the control and treated cells as described above) were collected and stored at − 80 °C. The supernatants were diluted two fold and albumin levels determined by Human serum albumin DuoSet ELISA according to the manufacturer’s instructions (Bio-techne, USA).

### Immunohistochemistry, brightfield and enhanced darkfield imaging of NM distribution

Following the final exposure to NMs and DQ12, the MTs were harvested washed once with 1x PBS, and fixed in 4% paraformaldehyde overnight. The fixed spheroids were embedded in Eppendorf tubes with 2% agarose and further processed for paraffin embedding and sectioning of 4–5 μm followed by standard haematoxylin and eosin (H&E) staining. Next, a Cytoviva enhanced darkfield hyperspectral system (USA) was used for detection of NMs in the cross section of the MT. Enhanced darkfield depictions were prepared by the acquisition of multiple overlapping images at 100x magnification, using an Olympus BX 43 microscope with a Qimaging Retiga 4000R camera. Overlapping images were assembled in ImageJ using the MosaicJ plugin [22, 23]. The brightfield images were acquired at 100x via an Olympus BX 43 microscope with a Nikon DS-Fi2 camera.

### Rotational visualisation of MT surface

In an attempt to visualise the cell/material interactions on the surface of the spheroids, the MT were treated with 25 μg/ml of a 200 nm fluorescently linked microsphere polymer bead (Life Technologies, UK) for 24 h. The MT was fixed as described above and re-suspended in Mowiol®4–88 mounting medium (Sigma-Aldrich, UK) and loaded into glass capillaries (one MT per capillary with internal diameter of 0.86 mm and outer diameter of 1.2 mm - Harvard apparatus, UK). The glass capillaries were attached to a manual rotation stage using a v-groove holder (Thorlabs, UK) (for precise rotation), and mounted in a custom stage on an inverted microscope (Motic, Germany). Brightfield and fluorescent images (365 nm excitation) were acquired using a 40x air objective (Olympus, UK) using a Canon 650d camera (Canon, UK).

### Statistical analysis

The data are expressed as mean ± standard error of the mean (SEM). For statistical analysis, the experimental results were compared to their corresponding control values using full-factorial ANOVA with Tukey’s multiple comparison. All statistical analysis was carried out utilizing Minitab 18. A *p* value of < 0.05 was considered to be significant. The experiments were repeated a minimum of three occasions (unless otherwise stated).

## Results

### Characterisation of pristine and dispersed NMs

A list of the main measured physiochemical properties for the NMs has been described previously [18–20] and reproduced in Table 2. Furthermore, in order to investigate NM behaviour in the liver maintenance medium, the hydrodynamic size distribution of the materials was investigated (Table 2). In addition, no endotoxin contamination (≤ 0.25 EU/ml) was detected in any of the NM suspensions.

**Table 2:** Main physical and chemical properties of investigated NMs (adapted and reproduced from 18 to 20)

NM code	NM type	Phase	Primary size (nm) (XRD)	Surface area [m^2^/g] (BET)	Known coating	Mean size in liver maintenance medium (DLS) (nm)^a^
JRCNM01005a	TiO_2_	Rutile-anatase	15–24	46	None	224.6 233.7 238.3
JRCNM01101a	ZnO	–	152	15	Triethoxy-caprlsilane	250.3 255.8 255.4
NM212	CeO_2_	Irregular and non-homogeneous - cubic cerionite	49	27	None	274.5 269.3 258.9
DQ12	Silica	Quartz	–	10.1	None	479.8 468.5 466.9

*DLS* Dynamic light scatter, *XRD* X-ray diffraction

^a^Size in biological media measured within 30 min of sonication

### NM-induced cell cytotoxicity - AK assay

**Fig. 1:**
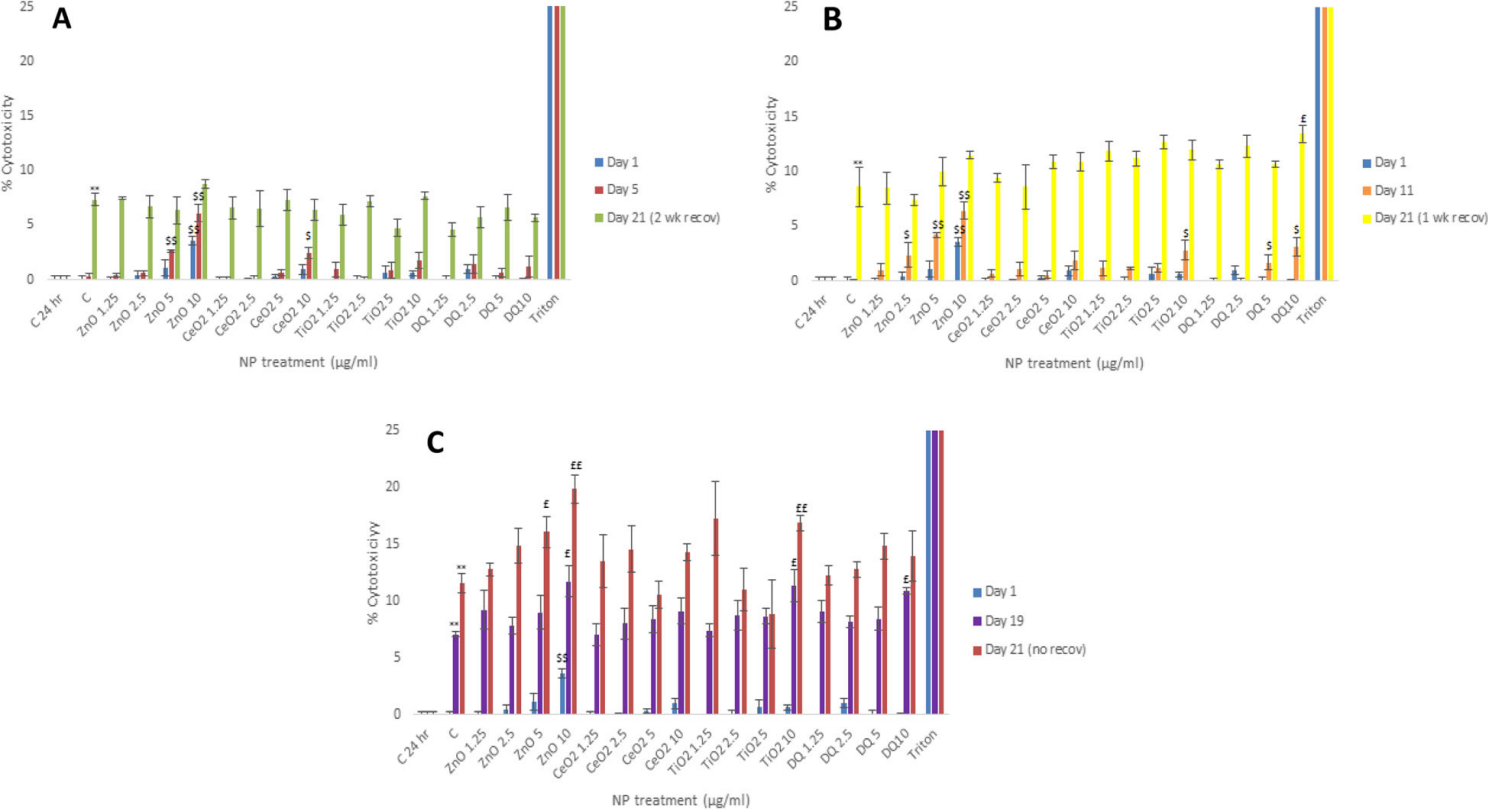
Cytotoxicity in human liver MT following exposures to a panel of engineered NMs for up 3 weeks as measured by AK release via ToxiLight™ cytotoxicity assay (NM treatments 1.25–10 μg/ml). **a** Experiment A - 7 days of NM dosing following 2 weeks of recovery; **b** Experiment B - 14 days of NM dosing following 1 week of recovery and **c** Experiment C - 21 days of NM dosing with no recovery period. The Triton data depicts 100% cell death ± SEM. The values represent mean ± SEM (*n* = 3) with significance indicated by * *p* < 0.05 and ** *p* < 0.005 for ageing effects compared to day 1 negative control, $ *p* < 0.05 and $$ *p* < 0.005 signifying NM induced and £ *p* < 0.05 and ££ *p* < 0.005 NM-induced effects in addition to the ageing effect

The AK data showed that generally speaking, the observed cytotoxicity was relatively low in these experiments with cell death never increasing above 20% over the 3 weeks of cell culture regardless of the exposure regime (Fig. 1a-c). Interestingly, a significant ageing effect was observed with up to 12% cell death in the negative control MT after 3 weeks of cell culture (Fig. 1c). It is important to state that the cell death in the control MT only became apparent after 19 days of culture (~ 8% as compared to the negative controls at day 1) (Fig. 1c). Furthermore, the data demonstrated that the NM-induced cell death at the utilized concentrations was small with only diminutive differences notable between the different NM types. The most consistent NM-induced toxicity was attributed to the ZnO with the effects being dose and time dependent (~ 8% higher than control - values representative of NM-induced death above the aging effects). TiO_2_ NM also induced cytotoxicity at the highest concentration and the latest time-point (6% higher than control respectively - induced death above the aging effects) (Fig. 1c). Overall, we suggest that the demonstrated approach of low dose repeated long-term exposure regimes might be most physiologically relevant and “realistic” strategy for hepatic nanotoxicology, which might allow for in vivo comparisons to be made (Kermanizadeh *et al.* 2019 A review of hepatic nanotoxicology - considerations for the next generation of study designs. Under review in Particle and Fibre Toxicology, Kermanizadeh *et al.* 2019a and 2019b - manuscripts in preparation). This issue is further explored in the Discussion section.

### Analysis of AST as a biomarker of liver toxicity

Due to the fact that AST levels are routinely utilized as a bio-marker of liver damage in clinical settings and a routinely investigated in vivo parameter two different methodologies (Bioanalyser and ELISA) were utilized to ascertain the feasibility of this particular toxicological end-point in the detection of potential NM-induced cell damage in vitro. Unfortunately, neither method was sensitive enough to detect the potentially small NM-induced fluctuations in AST activity (despite up to 20% cell death detected in the AK assay). This being said, both methods were validated by the inclusion of the positive control (triton) causing a significant increase in AST levels as compared to the negative controls (data not shown). Overall, the data suggests that AST measurements might not be a suitable end-point for detection of NM-induced hepatic cell death in vitro, especially taking into account the fact that the adverse effects observed in this study are not of acute nature but rather gentle and over time.

### Impact of engineered NMs on albumin production by human liver MT

**Fig. 2:**
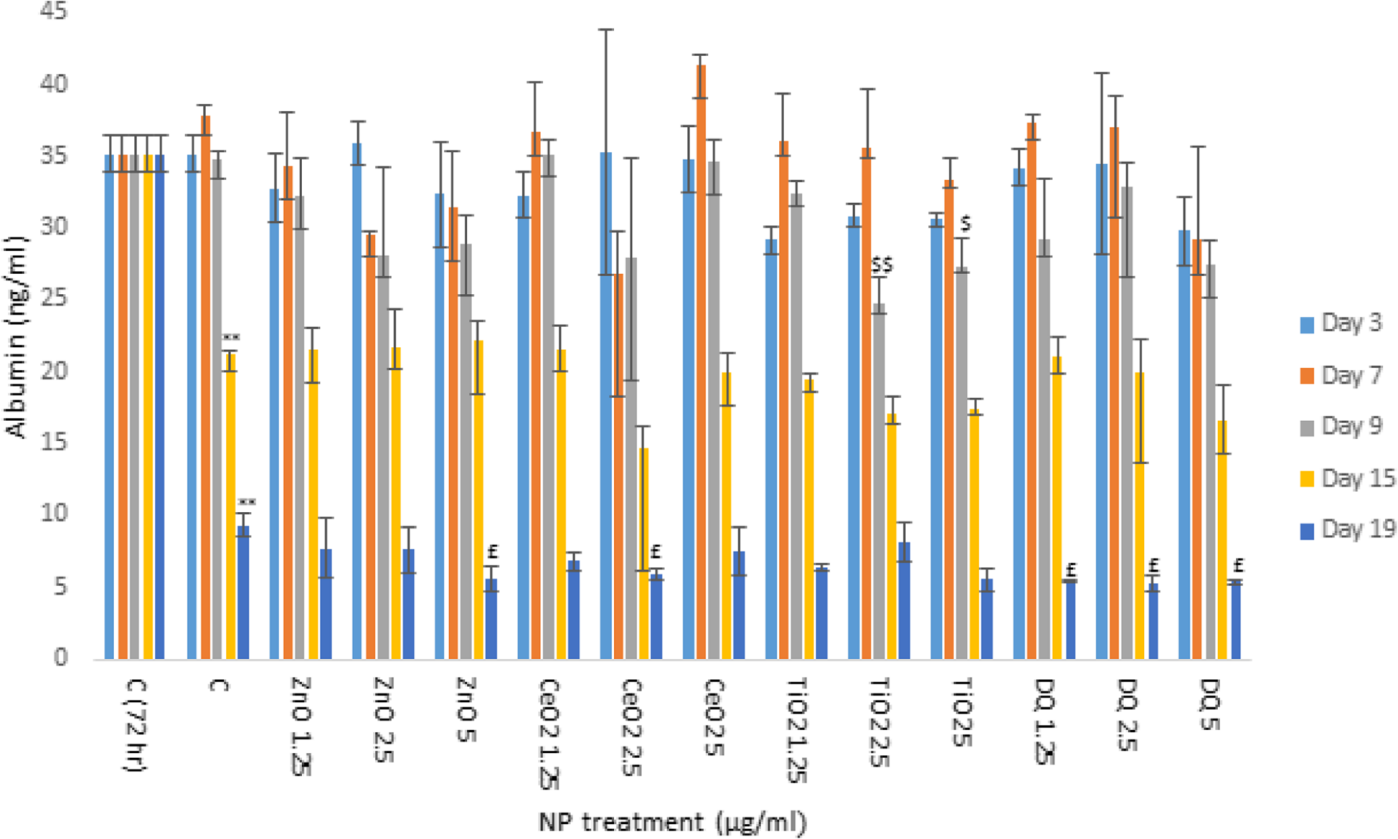
Albumin production from human liver MT following exposure to a panel of engineered NMs (NM treatments 1.25–5 μg/ml) for up 19 days. The values represent mean ± SEM (*n* = 3) with significance indicated by * *p* < 0.05 and ** *p* < 0.005 for ageing effects compared to 3 days negative control, $ *p* < 0.05 and $$ *p* < 0.005 signifying NM-induced and £ *p* < 0.05 and ££ *p* < 0.005 NM effects in addition to the ageing effects

Next, NM effects on albumin production (as a marker of hepatocyte function) was investigated over a 19 day exposure period (Fig. 2). The data clearly showed a large and significant ageing effect with a clear reduction of albumin production in the control MT as early as at 15 days of cell culture. Overall, the NM-induced effects appeared small. For TiO_2_ NM exposure at concentrations of 2.5 and 5 μg/ml caused the most significant decrease compared to control at day 9 (Fig. 2). In addition, small yet statistically significant alterations were noted for the higher concentrations of ZnO and CeO_2_ at day 19 as well as for DQ12.

### Cytokine secretion from liver MT following NM exposure

**Fig. 3:**
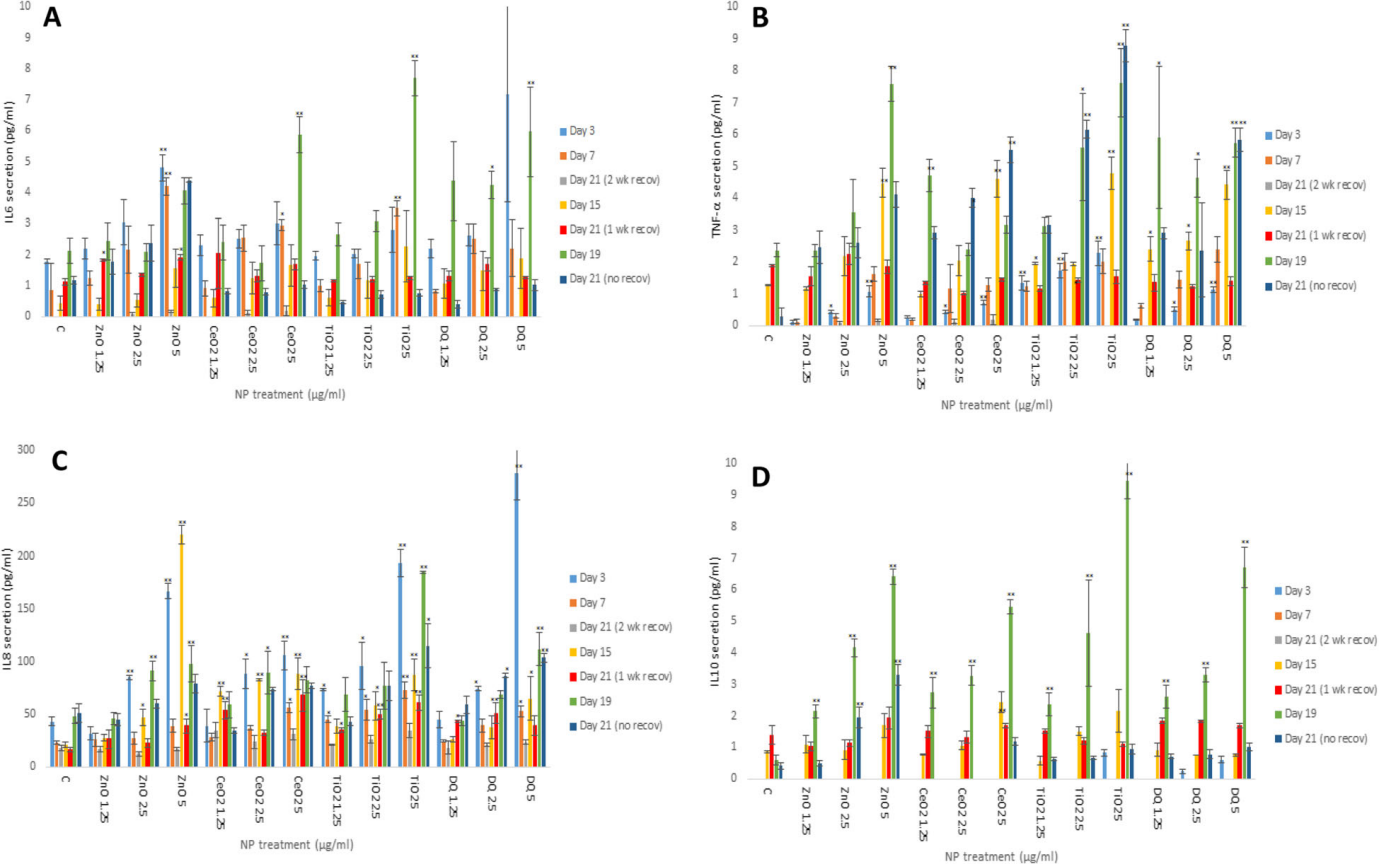
IL6, TNF-α, IL8 and IL10 levels from NM exposed human liver MT. The tissues were exposed to cell medium (c) or repeated concentrations of NMs (NM treatments 1.25–5 μg/ml) for up to 3 weeks - **a** IL6, **b** TNF-α, **c** IL8 and **d** IL10. The values represent mean ± SEM (*n* = 3) with significance indicated by * *p* < 0.05 and ** *p* < 0.005 of NM-induced effects compared to negative control

The alterations in cytokine production levels (TNF-α, IL6, IL8 and IL10) as a consequence of NM exposure was assessed within the supernatant of control and NM-exposed liver MT (Fig. 3). Generally speaking, it was difficult to ascertain clear patterns for cytokine secretion following exposure to the different NMs. However, the in depth analysis of the data highlighted two clear and important observations. Firstly, the two recovery periods proved to be sufficient for a reduction in the inflammatory protein secretion levels as compared to 7 days and 15 days (two-week or one-week recovery periods respectively). Secondly and interestingly, the anti-inflammatory IL10 response became detectable at day 15, with the levels of protein produced increasing up to day 19 (no recovery) (Fig. 3d). Other observations included increases in ZnO NM-induced IL6 levels at day 3 and 7 which reduced following recovery periods up to day 21 (experiment A - Fig. 3a). TNF-α secretion was most notable at the higher NM doses. In Experiment A, significant (albeit small) TNF-α secretion was detected with these levels dropping following recovery periods up to day 21. In experiment B, significant TNF-α levels were noted following exposure to higher doses of all NMs which again dropped following recovery periods (Fig. 3b). As expected, in Experiment C significant and highest TNF-α levels were observed at day 19 and 21 following exposure to all NMs (most evident for ZnO and TiO_2_ NM). For IL8, in experiment A statistical significance was noted at day 3 but not day 7 or 21. In experiment B, significant increases were detected at day 15 but not 21. Finally, in experiment C significant IL8 levels were observed at day 19 and 21.

### Brightfield and enhanced darkfield imaging of NM distribution within the MT

**Fig. 4:**
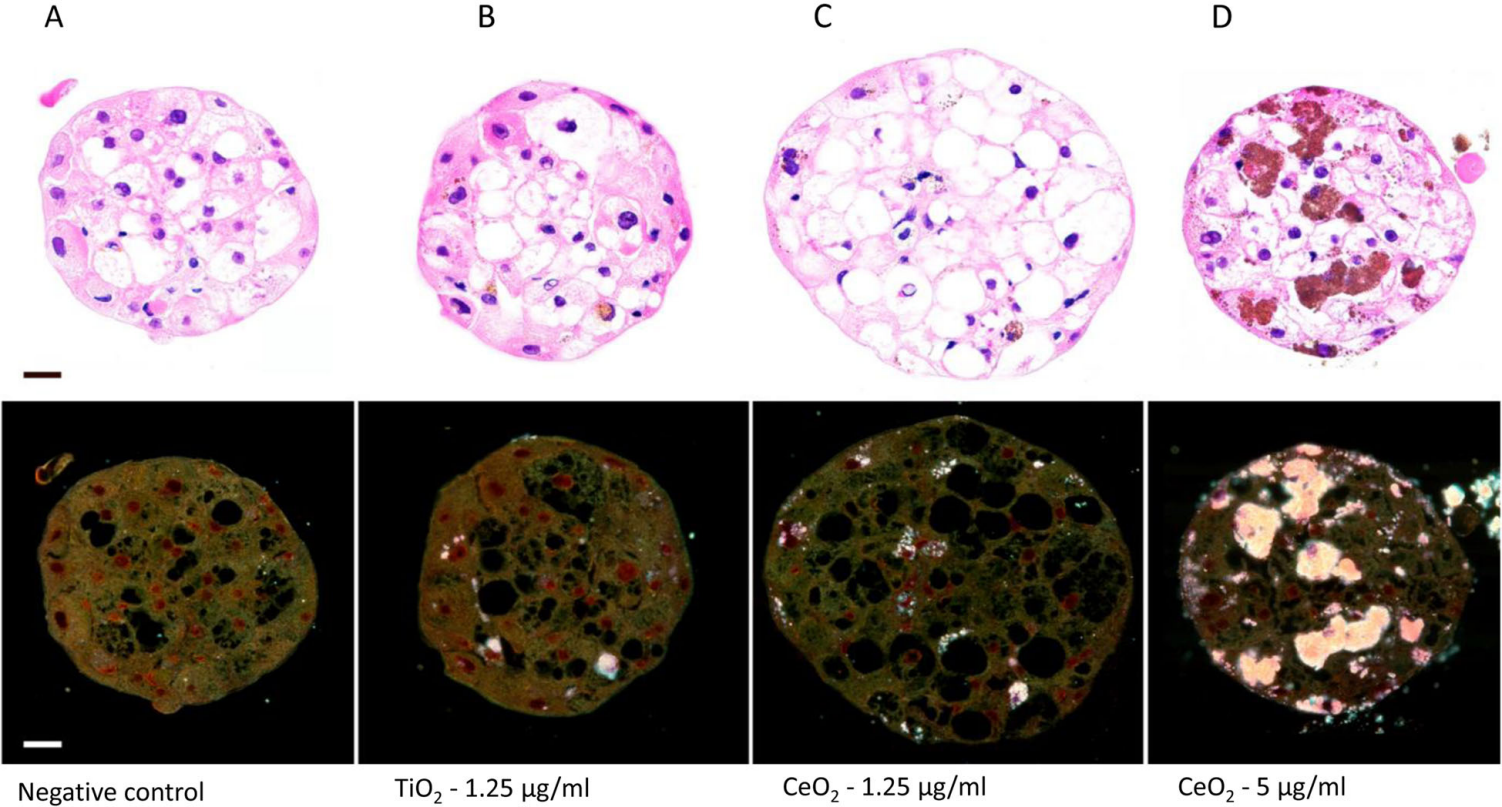
Representative brightfield and enhanced darkfield images of the distribution of NMs in the multi-cellar primary human liver MT after 3 weeks of cell culture - **a** negative control, **b** TiO_2−_ 1.25 μg/ml, **c** CeO_2_–1.25 μg/ml and **d** CeO_2_–5 μg/ml. H&E stained cross sections, Scale bar - 20 μm

Due to the tightly packed three-dimensional structure of the spheroids, it was imperative to investigate whether the inner cells in the MT come in direct contact with low solubility NMs or whether the biological response was solely governed by the cells on the outer layer. The brightfield and enhanced darkfield imaging of cross-sections of TiO_2_ and CeO_2_ NM treated MT showed conclusively that the NMs penetrated deep into the MT (Fig. 4 and Table 3). Hence, it is safe to presume that a significant proportion of the cells come into contact with the NMs over the repeated exposure regime. Another unintentional yet interesting observation included the detection of numerous steatotic hepatocytes (fat accumulation inside the hepatocytes) after 3 weeks of cell culture (equally present in the controls and the NM treated MT). This finding, might partially account for the ageing effects detected via the other toxicological end-points (viability and albumin production).

**Table 3:** Incidences of liver MT with internalized CeO_2_ and TiO_2_ NM at 3 weeks

MT exposure	Representative image in Fig. 4	MT with internalized NMs	Notes
Negative control	a	(1)/13	Small agglomerate detected in one of the 13 MT investigated. Due to the fact, that the material was not present in any of the other sections, it is considered as an artefact.
TiO_2_–1.25 μg/ml	b	5/5	
TiO_2_–5 μg/ml		2/2	
CeO_2_–1.25 μg/ml	c	8/8	
CeO_2_–5 μg/ml	d	8/8	

### Rotational visualisation of MT surface

**Fig. 5:**
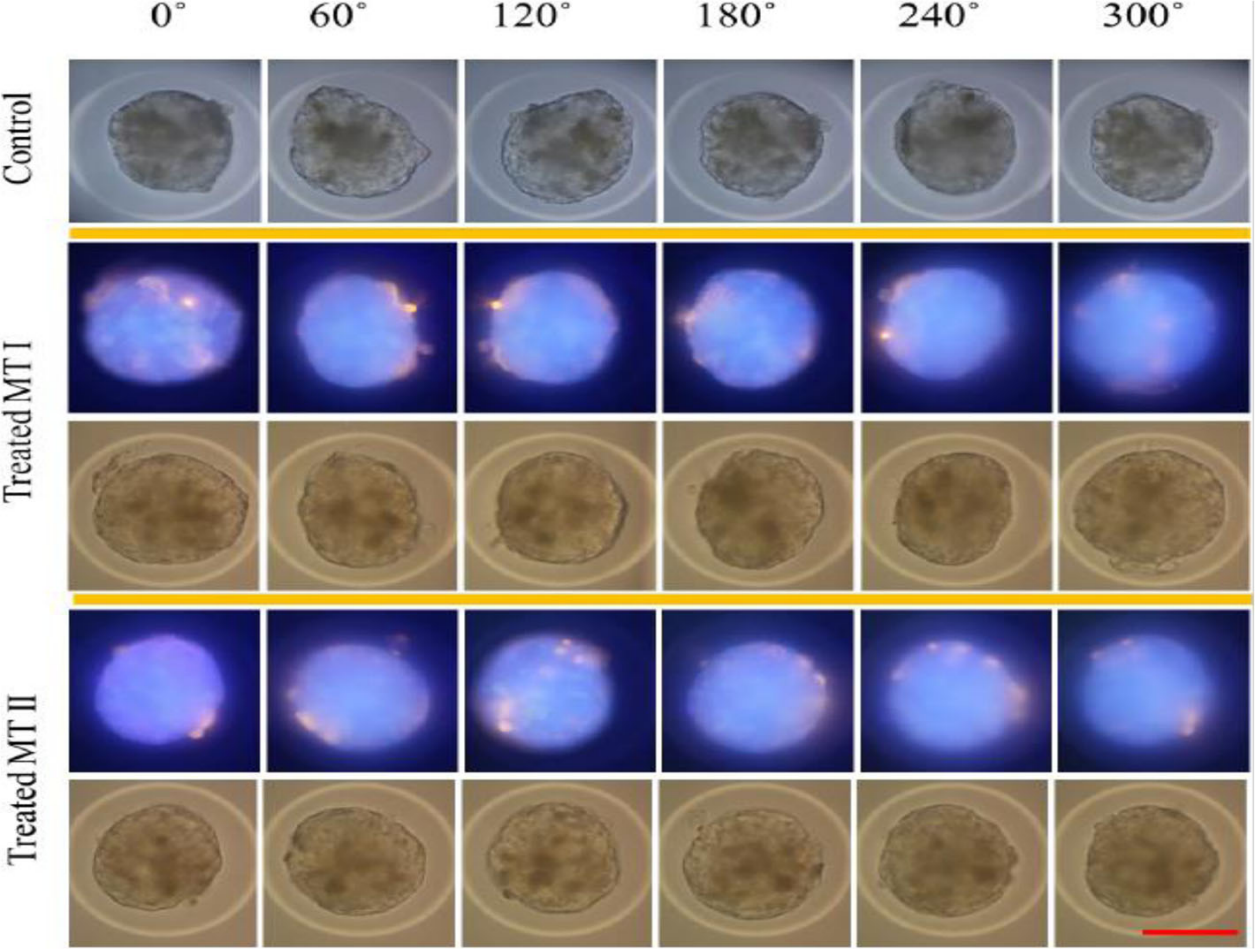
The surface visualisation of MT treated with fluorescently linked polymer microspheres at 24 h. The scale bar corresponds to 200 μm and in the fluorescent images, orange colour indicates presence of fluorescent polymer microspheres and blue is an effect of autofluorescence

Finally, the interaction of materials with the surface of MT was investigated. This was an important consideration due to the fact that the spheroid MT are not attached to the bottom of the wells. Therefore, there was a necessity to ascertain whether the MT rotate in the wells and if the NMs encounter the cells equally on all surfaces (mostly observed as aggregates). MT treated with the microspheres in the culture wells were fixed in order to preserve NMs localisation on their surface. Next, they were transferred to mcirocapilaries containing mounting medium and NMs distribution was visualised in 360 degree view by rotating them. The rotation and visualisation of the material exposed MT showed even distribution over the full surface of the spheroid (Fig. 5 and Supp Fig. 1). The data suggests that the MT might rotate in the well and the exposure is not restricted to the apical surface of the MT (i.e. the rotation in the wells might suggest that that there is not really an apical surface for the exposures).

## Discussion

This unique study was designed to assess the suitability of a multi-cellular primary 3D liver MT model for long-term in vitro nanotoxicological assessment. The MT offers a functionally and metabolically active system in a 96 well plate format with no scaffolds or hydrogels that utilises primary human hepatic cells. The lack of scaffolds or hydrogel is very important for particle toxicology due to the fact that such physical barriers could impede cell and NM interaction. Additionally, the model allows for one MT per well with a defined cell number. The liver cells in this model are viable for long periods, which allows for low dose multiple particulate treatments over a period of weeks. This might equate to more physiologically relevant and realistic exposure scenarios making them potentially useful for investigating hepatic toxicity and disease progression in vivo.

In order to achieve our aims, the MT were exposed to a panel of four NMs with different physicochemical characteristics (ZnO, CeO_2_ and TiO_2_ NMs and crystalline silica). The toxicological effects of the panel of NMs was assessed on the human liver MT following 3 weeks of low dose repeated exposures, which included recovery periods of 1 or 2 weeks. It was our intention to avoid NM-induced cell death and focus on the toxicological assessment at sub-lethal concentrations (the rationale for this decision will be discussed in the upcoming section). The data from the study showed that material-induced cell death was very low, even after ten repeated exposures for up to 3 weeks. However, up to 12% cell death was observed in the negative control MT after 3 weeks of cell culture, which is an important consideration for future experimental design. Interestingly, there was no cell death in the medium treated MT at 2 weeks. This suggests that 2 weeks of cell culture might be close to limits before subtle ageing effects start to overshadow low sub-lethal NM-induced cellular responses in this test system.

Next, NM-induced fluctuations in AST levels were analysed at the 3 week time-point. In this study, AST levels were regarded as highly important due to the fact that they are regularly measured clinically to appraise liver damage; as well as being routinely analysed in in vivo experimentations. Significantly, the possibility of measuring the same bio-marker of damage both in vitro and in vivo was very appealing. However, none of the two methods utilized within in this study were sensitive enough to detect the potentially subtle NM-induced fluctuation in enzyme activity. Therefore, we advocate that in vitro AST measurement are not currently suitable in a nanotoxicological context. It might be important to state that generally speaking NM-induced effects on AST activity in vivo have only been reported following use of high doses.

TNF-α is one of the main contributors to septic shock. It stimulates the release of corticotropic releasing hormone, which induces fever, and suppresses appetite. Furthermore, it also stimulates the acute inflammatory response by promoting the synthesis of C-reactive protein, serum amyloid A and other mediators in the liver. The cytokine also induces vasodilation and vascular permeability both critical for leukocyte infiltration [24]. TNF-α is also important in the recruitment of immune cells to the inflammation site [25]. IL6 is an important mediator of innate immunity. The protein is secreted by macrophages; and instigates various innate immune signalling cascades resulting in an augmentation of inflammatory responses and cell recruitment [26] Furthermore, the cytokine is responsible for stimulating acute phase response protein secretion from the liver as well as the production of neutrophils [27, 28]. IL8 is a potent chemokine, mediating the activation and migration of a wide variety of inflammatory cells and play a pivotal role in initiation of an inflammatory response [29, 30]. IL10 is a multi-functional cytokine with diverse effects on a wide range of cells. The cytokine’s principle function is to terminate inflammatory responses [31, 32]. The cytokine also plays a crucial role in the differentiation of regulatory T cells, which are involved in the control of inflammatory responses and development of immune tolerance [32].

The analysis of the pro/anti-inflammatory protein secretion showed time dependent NM-induced increases in cytokine levels. It was difficult to detect a clear difference for cytokine secretion between the four different materials. The cytokine analysis proved useful in highlighting recovery periods as being sufficient for allowing a reduction in the pro-inflammatory response. The data showed a significant anti-inflammatory response from the MT at later time-point, which adds credence to our previous observations of organ specific immune tolerance being favoured in a healthy liver following an acute NM challenge [13].

The final toxicological end-point showed NM-induced reduction in albumin production to be small; with, TiO_2_ NM exposure at concentrations of 2.5 and 5 μg/ml causing the most significant decrease at day 9. In addition, small yet statistically significant alterations were observed following exposure to ZnO, CeO_2_ and DQ12 at day 19. This was the first instance in which we have observed sub-lethal NM induced reduction in albumin levels (no reduction in albumin production was noted in previous in vitro experiments - [20, 33-35]). These differences might be explained by the long-term, repeated exposures utilized in this study, which go beyond anything attempted previously. The results also showed a large ageing effect with a clear reduction in albumin production from the hepatocytes in the control MT at day 15 of cell culture.

Next, brightfield and enhanced darkfield imaging of the paraffin embedded MT cross-sections of low soluble NM-treated MT showed a concentration-dependent penetration of materials deep into the tissue. The data also demonstrated a large number of steatotic hepatocytes after 21 days of cell culture. Finally, the investigation of the interaction of a fluorescently labelled particles with the surface of MT suggested that the MT might rotate in the well and the exposure is not restricted to a specific apical surface of the tissue.

It is difficult to compare the data generated within this study to previous work, as this work is novel both in terms of test model and the dosing strategy utilized yet there is some accordance to our previously published work in particular with regards to the inflammation end-points most notable with regards to ZnO induced pro/anti-inflammatory response [34, 35]. Of course the materials ZnO and TiO_2_ differ considerably in terms of their solubility, with ZnO being more soluble [20]. Within the body, soluble components have the potential to have local effects, but also to be cleared from the site of particle deposition through movement of tissue, lymphatic and plasma fluids. The model used in this paper included static culture conditions. Future models should consider inclusion of flow with such models in order to better understand the differences between materials with different dissolution rates.

The main aim of this study was to assess the suitability of the complex hepatic test system as a suitable surrogate for in vivo experiments. From the scrutiny of the in vivo toxicology literature [Kermanizadeh et al. 2019 A review of hepatic nanotoxicology - considerations for the next generation of study designs. Under review in Particle and Fibre Toxicology] it is clear that to date any NM-induced adverse effects observed in the liver at acute time points, have the potential to resolve. In almost all studies in which a recovery period was included the healthy liver was able to recover from the NM challenge (irrespective of NM type, toxicological end-point investigated, dose or route of exposure) (i.e. [36-38]). This being said, a considerable body of in vivo data demonstrate NM accumulation in the liver (predominately in the KCs) for periods of up to 1 year post exposure (i.e. [17, 39, 40]) which is not necessarily associated with adverse effects in the liver. It is not inconceivable to hypothesise that in man, any accidental NM exposure scenario that might have a “realistic” hepatic hazard potential in all reality would only occur after long-term exposure. Therefore, it is essential to establish more advanced, physiologically relevant in vitro assessment tools for improved prediction of the adverse effects caused by life-time NM exposure in humans. To this end and for the reasons discussed above, the suitability of 24 h single exposure in vitro monocultures of hepatocytes for hazard assessment are questionable. Therefore, this hepatic nanotoxicology study was designed to take into account two imperative organ specific principals:

1. Of great importance and unique for the liver, is the organ’s incredible regeneration capability. The livers ability to regenerate is critical in disease recovery and in distinguishing the ability of different NMs to induce longer-term harm to the human liver. This consideration should always be considered in NM hazard assessment strategies. The healthy incredible liver’s ability to regenerate cannot currently be replicated or reproduced in vitro*.* Therefore, it stands to reason that meaningful in vitro nanotoxicological data might only be possible by utilisation of very low (sub-lethal) repeated long-term dosing regimens.
2. In vitro acute cytotoxicity (measured as cell death or viability) measurements alone are not beneficial for hazard ranking of NMs in the liver, as such measurements have little in vivo relevance and the findings do not relate to in vivo observations. Therefore, the selection of organ specific sub-lethal toxicological end-points are more meaningful for predication of “real” NM-induced in vivo hazard.

To this end, this study was only a partial success. As described above we clearly demonstrated that the utilisation of low dosing strategies is more than sufficient to prompt NM-induced biological effects. The toxicological changes increased with both concentration and time. Moreover, the utilisation of repeated low dosing highlighted the complexities of cytokine biology in the liver both in terms of differing epochs of individual cytokines as well as demonstrating the fine balance of pro vs. anti-inflammatory cytokine counteractions. We identified albumin secretion as a potential useful toxicological end-point following low dose repeated dosing in vitro. This being said, due to albumin being one of the most abundantly present proteins in man (and rodents) it is unlikely that administration of NMs at physiologically relevant doses would cause significant fluctuations that would be measurable as a bio-marker of NM-induced liver toxicity in vivo (i.e. [41]).

Despite the above-described successes, the study (and test model) falters in identifying a NM-induced toxicological endpoint that is measureable both in vitro and in vivo*.* The two methodologies utilized to measure AST activity were not sensitive in identifying potentially low fluctuations. Furthermore, generally speaking, the recovery periods were not all that efficacious due to relatively large ageing effects in the final week of these experiments. Overall, the advantages and pitfalls of the multi-cellular primary 3D liver MT are highlighted in Table 4.

**Table 4:** The main nanotoxicological advantages and disadvantages of multi-cellular primary 3D liver MT in the assessment of the tissue as a potential in vivo surrogate

Advantages	Disadvantages
Human tissue	Cost of plates can be potentially prohibitive to academia
Primary cells	Toxicological assessment beyond 2 weeks might not be ideal in a nano context
Incorporation of numerous liver cell populations - hepatocytes, Kupffer cells and endothelial cells	There is no physiological structure to the tissue - cells aggregate randomly
High metabolic activity	
In vitro model which allows for repeated long term exposure of xenobiotics	
Little variability between MT in wells and plates	
Scaffold-free with 100% endogenous extracellular matrix	

Our future experiments will attempt to address the following important issues:

1. Attempt to quantify NM uptake over time
2. Tissue clearance and specific cell staining will be utilized to visualise the interaction/uptake of materials by the different cell populations (most importantly KCs) in the MT. This is upmost importance as the KC in vivo are localized within the lumen of the sinusoid. Due to their locality they are the first and most important cell population that encounter non-soluble particulates reaching the organ (i.e. [42, 43]). In addition, these cells govern the immune response in the organ in a healthy liver.
3. Future experiment will only continue to a maximum of 2 weeks (to avoid ageing effects) in order to ascertain whether meaningful MT recovery from a NM challenge is feasible.
4. A number of candidate biomarkers of liver damage will be investigated in an attempt to establish NM-stimulated toxicological changes that are detectable both in vitro and in vivo which will hopefully allow for direct and more meaningful comparisons between the data sets.
5. The healthy MT will be modified to improve the pathophysiological relevance - diseased MT will be representative of steatosis (mild damage) and fibrosis (severe damage). It has been demonstrated that NM-induced adverse effects in the liver are significantly aggravated in the diseased organ [41, 44]. Additionally, the disease state of the liver might influence and hamper the organ’s ability for regeneration post-NM challenge. As an important and additional complication, a large number of the general population suffer from a wide spectrum of sub-clinical liver damage without any apparent visible disease manifestations. Therefore, it is critical that a range of liver diseases (mild to severe) are considered in hazard and risk assessment strategies for NMs.

## Conclusion

With the advances in the fields of nanotechnology and nanomedicines the potential of nanotoxicological effects on the liver is an unavoidable reality. As a tool for better understanding of “real” NM-induced hepatic hazard we believe that the 3D multi-cellular primary human liver MT utilised within this study to be a strong candidate; something which might not be necessarily possible when using single exposure monocultures of hepatocytes.

## Supplementary information

**Additional file 1: Figure S1.** The size distribution of the four different materials in human liver maintenance medium as measured by Dynamic Light Scattering using a Zetasizer Nano-ZS. **Figure S2.** Exemplary fluorescent image of untreated control non-treated MT showing blue autofluorescence. The scale bar corresponds to 200 μm.

## Data Availability

Not applicable.
