# Supplementary figures and images for: Assessment of nanomaterial-induced hepatotoxicity using a 3D human primary multi-cellular microtissue exposed repeatedly over 21 days - the suitability of the in vitro system as an in vivo surrogate
Source: Part Fibre Toxicol. 2019 Nov 19;16:42. doi: 10.1186/s12989-019-0326-0 (PMC6862829; doi:10.1186/s12989-019-0326-0)

## Slide 1
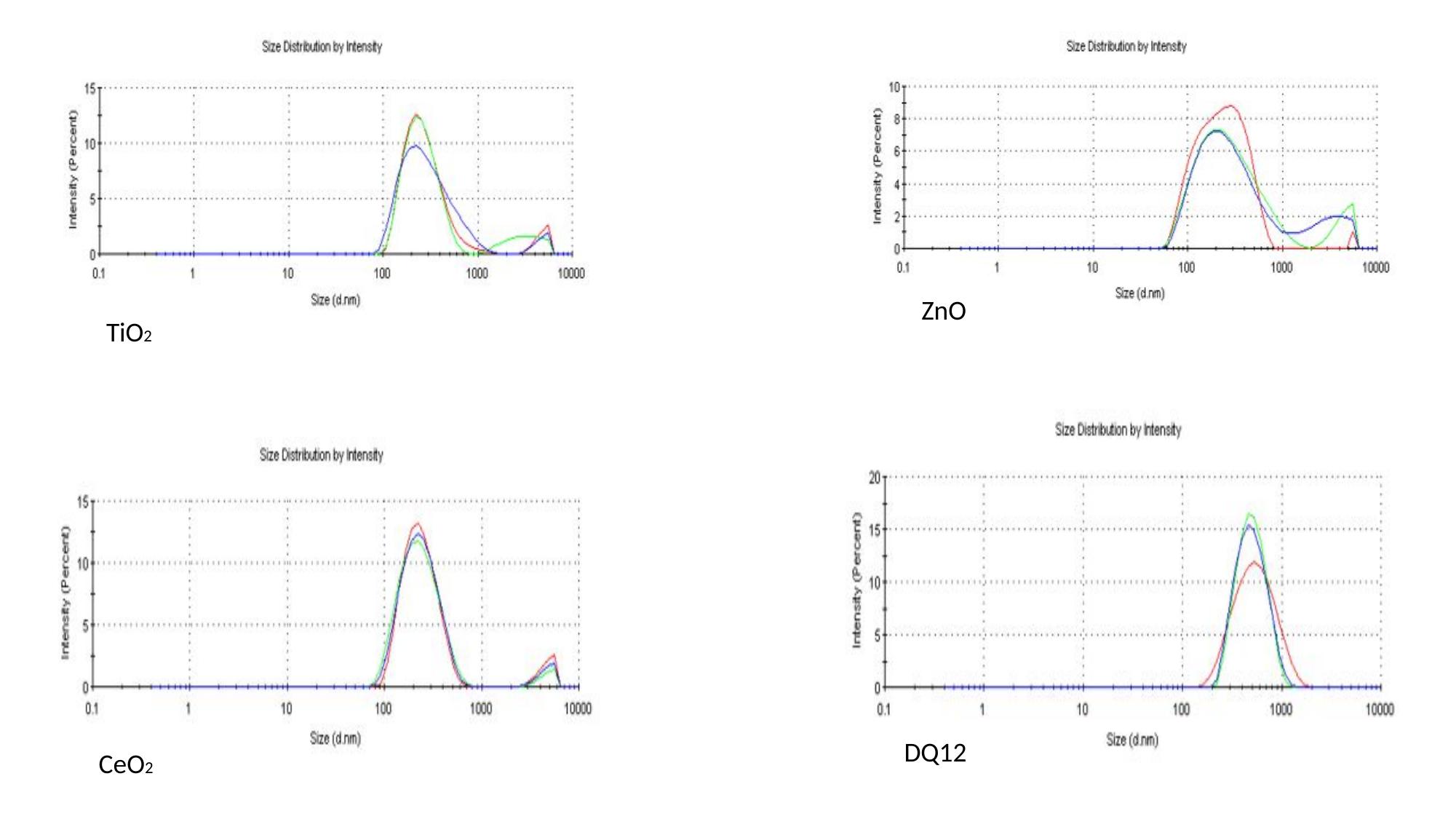

ZnO
TiO2
DQ12
CeO2

## Slide 2
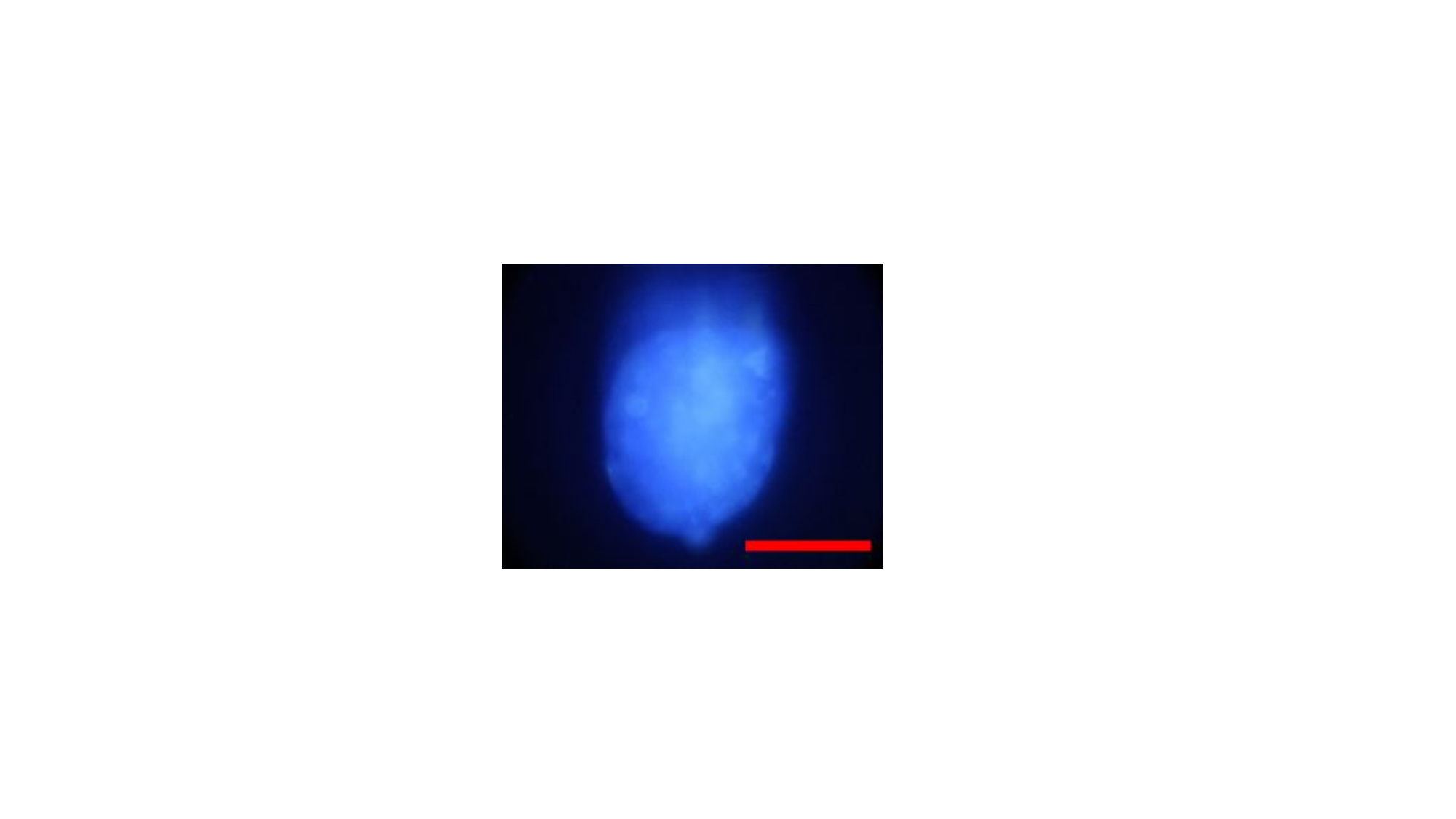

#

Supplement: Supplementary file 1 — Additional file 1: Figure S1. The size distribution of the four different materials in human liver maintenance medium as measured by Dynamic Light Scattering using a Zetasizer Nano-ZS. Figure S2. Exemplary fluorescent image of untreated control non-treated MT showing blue autofluorescence. The scale bar corresponds to 200 μm. [file 12989_2019_326_MOESM1_ESM.pptx]
